# YAP promotes tumorigenesis and cisplatin resistance in neuroblastoma

**DOI:** 10.18632/oncotarget.16209

**Published:** 2017-03-15

**Authors:** Chao Yang, Juan Tan, Jun Zhu, Shan Wang, Guanghui Wei

**Affiliations:** ^1^ Department of Pediatric Surgical Oncology, Children's Hospital of Chongqing Medical University, Ministry of Education Key Laboratory of Child Development and Disorders, Chongqing, China; ^2^ China International Science and Technology Cooperation Base of Child Development and Critical Disorders, Chongqing, China; ^3^ Chongqing Key Laboratory of Pediatrics, Chongqing, China; ^4^ Clinical Department of Children's Hospital of Chongqing Medical University, Lijia Campus, Chongqing, China; ^5^ Department of Pathology, Children's Hospital of Chongqing Medical University, Ministry of Education Key Laboratory of Child Development and Disorders, Chongqing, China; ^6^ Department of Urology, Children's Hospital of Chongqing Medical University, Ministry of Education Key Laboratory of Child Development and Disorders, Chongqing, China

**Keywords:** neuroblastoma, YAP, cisplatin resistance

## Abstract

The transcriptional co-activator Yes-associated protein (YAP) is essential for Hippo pathway-driven tumorigenesis in various cancers. However, the expression and function of YAP in neuroblastoma remains elusive. Here, we show that YAP was highly expressed in Neuroblastoma (NB) and expression levels correlated with advanced tumor staging. Knockdown of YAP significantly impaired neuroblastoma proliferation, tumorigenesis, and invasion *in vitro*. Injection of the YAP inhibitor, Peptide 17, dramatically prevented neuroblastoma subcutaneous tumor growth by efficiently downregulating YAP expression in tumors. Additionally, less proliferative and more apoptotic cells were found in the Peptide 17 treatment group. Furthermore, YAP inhibition significantly inhibited cisplatin-resistant neuroblastoma proliferation, tumorigenesis, and invasion *in vitro*. The combination of Peptide 17 with low-dose cisplatin efficiently impaired cisplatin-resistant NB subcutaneous tumor growth, being as effective as high-dose cisplatin. Notably, the combination therapy caused lesser liver toxicity in mice compared to the high-dose cisplatin treatment group. Collectively, this work identifies YAP as a novel regulator of neuroblastoma proliferation, tumorigenesis, and invasion and indicates that YAP is a potential therapeutic target for cisplatin-resistant neuroblastoma.

## INTRODUCTION

Neuroblastoma (NB) is a solid tumor that occurs in children and is characterized by a wide range of clinical manifestations and a poor prognosis [1]. After leukemia and brain/central nervous system tumors, NB is the third most common tumor affecting infants and young children [2]. NB accounts for 7% of all childhood malignancies but 15% of childhood cancer-related mortality, as over 50% of NB patients present with high-risk metastatic disease at the time of diagnosis [2]. In recent decades, advances in biology-based multimodal treatment strategies have led to an improved outcome for NB patients [3, 4]. However, the treatment also causes severe, long-term side effects including liver and renal functional lesion, deafness, cardiac failure, and secondary malignancies [3, 4].

Currently, most of the therapeutic strategies used in NB interfere with cell cycle progression and DNA synthesis or function, thereby causing DNA damage and the induction of apoptosis through the intrinsic and extrinsic apoptotic pathways [5, 6]. Cisplatin is one of the frontline chemotherapeutic drugs for NB and is widely used in clinical therapy [7]. Unfortunately, due to the acquired cisplatin-resistance of NBs, the prognosis of advanced NB patients after cisplatin treatment remains poor [8–10]. Thus, developing novel and efficient therapy targets is necessary.

The Hippo signaling pathway, which consists of Mst1/2, SAV1, Lats1/2, Mob, and Yes-associated protein (YAP), plays a crucial role in cell death, cell proliferation, and tissue growth [11–14]. YAP is the nuclear effector of the Hippo pathway that functions as an oncogene and is overexpressed in a variety of cancers such as hepatocellular carcinoma (HCC) [15], non-small cell lung cancer (NSCLC) [16], breast cancer [17, 18], sarcomas [19], colonic adenocarcinoma [20], gastric cancer [21], and lung adenocarcinoma [18]. YAP also participates in multidrug resistance of several cancers [22–24]. However, the expression and role of YAP in NB is still poorly understood.

In the present study, we sought to determine the expression of YAP in tissues from patients with NB. Further *in vitro* and *in vivo* solid experiments were performed to investigate whether YAP promotes tumorigenesis and maintains cisplatin resistance in NB.

## RESULTS

### High expression of YAP in NB is correlated with advanced tumor staging

**Figure 1:**
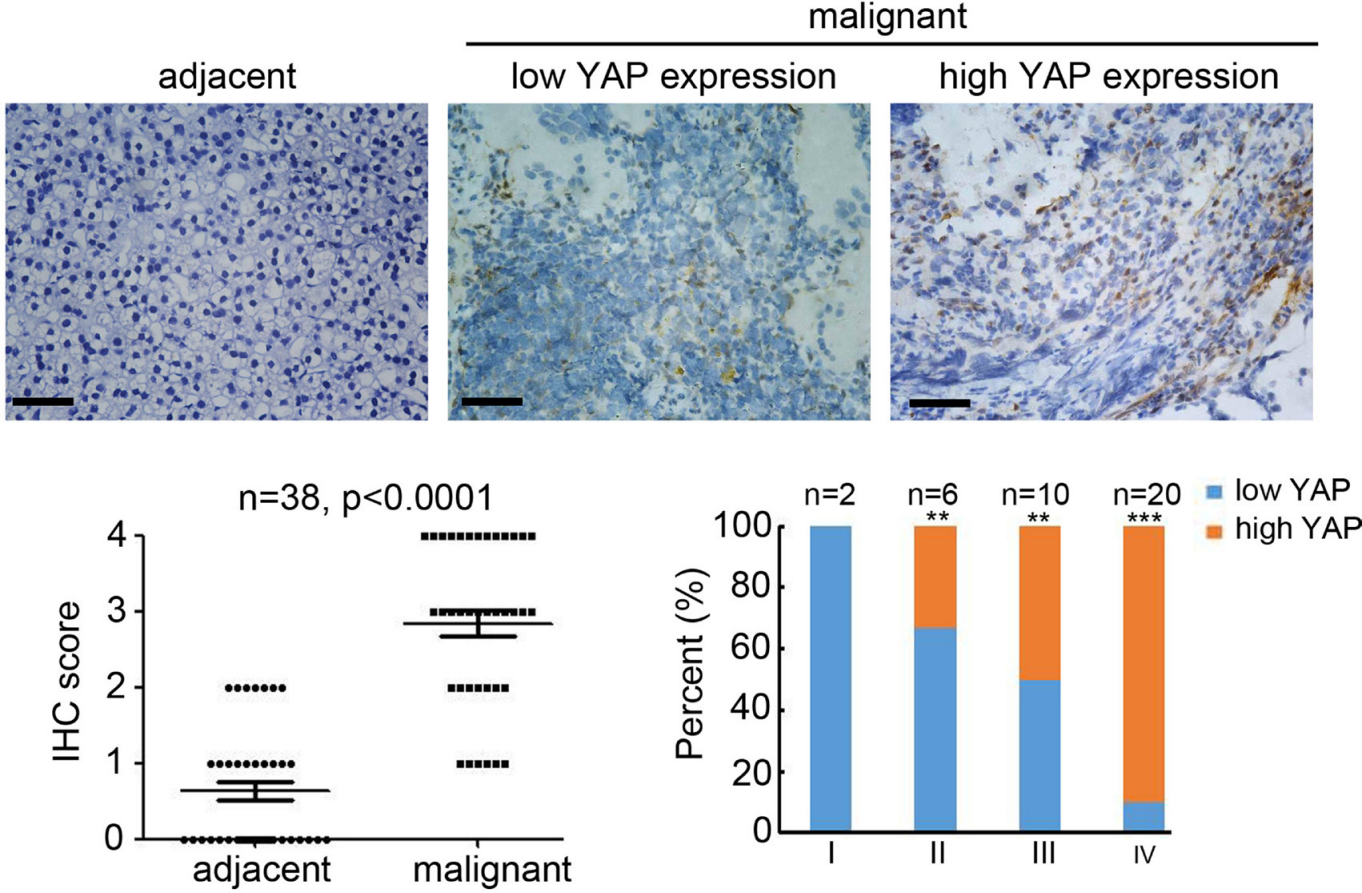
High expression of YAP in neuroblastoma correlated with tumor grade (**A**) staining for YAP on adjacent and malignant neuroblastoma tissues. Scale bar = 100 μm. (**B**) IHC score of YAP expression in normal and malignant neuroblastoma tissues (*n* = 30; **P < 0.01). (**C**) Percentage of patients with high and low expression of YAP according to tumor differentiation stage. (*n* = 38; **P < 0.01, ***P < 0.001).

To determine the potential predictive role of YAP in NB, we analyzed YAP expression in 38 malignant NB and adjacent tissues by IHC staining (Figure 1A). Compared with adjacent tissues, YAP expression in malignant NB tissues were significantly upregulated in 30 (78.9%) of 38 specimens (Figure 1A, 1B). Furthermore, higher YAP expression was detected in the stage III and IV tumors, whereas much lower YAP expression was observed in the stage I and II tumors (Figure 1C). Collectively, these results demonstrate that YAP expression is upregulated in malignant NB specimens and correlates with advanced tumor staging.

### Knockdown of YAP inhibits the proliferation and invasion of NB

**Figure 2:**
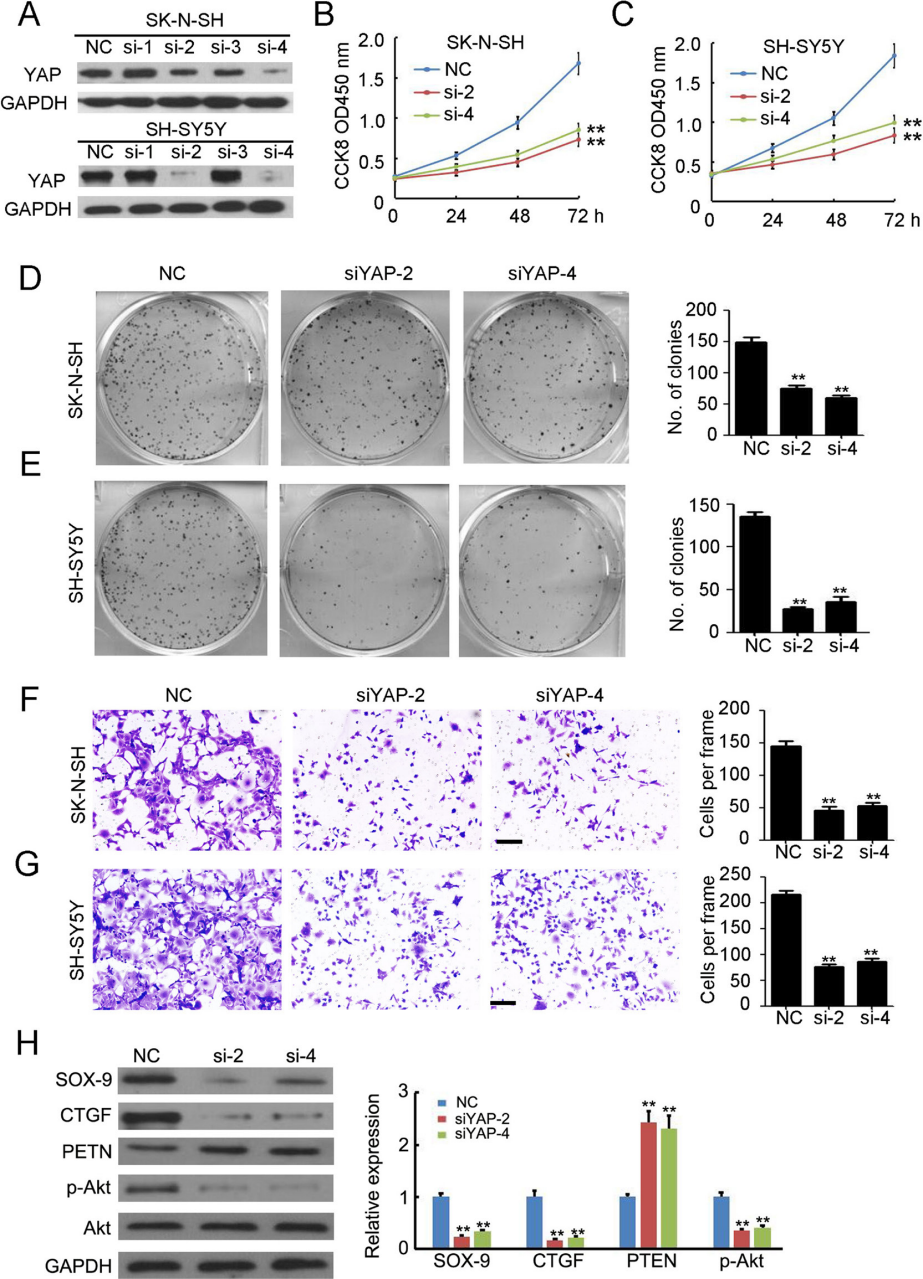
Knockdown of YAP inhibits the proliferation and invasion of neuroblastoma (**A**) four siRNAs targeting YAP were used to transfected SK-N-SH and SH-SY5Y cells. Total cellular extracts were prepared and subjected to western blotting using antibody against YAP. GAPDH was used as a loading control. (**B**, **C**) cell counting kit-8 assay was performed to determine cell proliferation of SK-N-SH and SH-SY5Y cells. Data represent the means ± SD from three independent experiments (**P < 0.01). (**D**, **E**) colon formation assay was performed to determine tumorgenesis of SK-N-SH and SH-SY5Y cells. Colony number was counted and analyzed. Data represent the means ± SD from three independent experiments (**P < 0.01). (**F**, **G**) transwell-mediated invasion assay was performed to determine the invasion ability of SK-N-SH and SH-SY5Y cells. The invaded cells in per frame were counted and analyzed. Data represent the means ± SD from three independent experiments (**P < 0.01). (**H**) detection of SOX-9, CTGF, PTEN, p-Akt and Akt expression in SH-SY5Y cells with or without siRNA transfection. GAPDH was used as a loading control. The relative expression was analyzed. Data represent the means ± SD from three independent experiments (**P < 0.01).

To explore the function of YAP in NB, we investigated the effects of siRNA targeted to YAP on cell proliferation, tumorigenesis, and invasion in cell lines expressing high levels of YAP (SK-N-SH and SH-SY5Y). Four potential siRNAs targeting YAP were employed to transfect SK-N-SH and SH-SY5Y cells, and western blotting results indicated that transfection of siYAP-2 and siYAP-4 efficiently inhibited YAP expression (Figure 2A). Knockdown of YAP significantly inhibited cell growth in both SK-N-SH (Figure 2B) and SH-SY5Y (Figure 2C) cells. Furthermore, fewer colonies were formed in the siYAP-2 and siYAP-4 transfected SK-N-SH (Figure 2D) and SH-SY5Y (Figure 2E) cells. Next, a Matrigel-mediated invasion assay was performed to determine the effects of YAP on NB invasion. We found that knockdown of YAP dramatically prevented SK-N-SH (Figure 2F) and SH-SY5Y (Figure 2G) invasion. To further investigate the mechanism responsible for these effects, several potential downstream targets of YAP were examined. The results indicated that knockdown of YAP significantly inhibited SOX-9, CTGF, and p-Akt expression while it induced PTEN expression. Taken together, YAP plays a critical role in regulating cell proliferation, tumorigenesis, and metastasis in NB cells.

### Peptide 17 inhibits NB tumor growth *in vivo*

**Figure 3:**
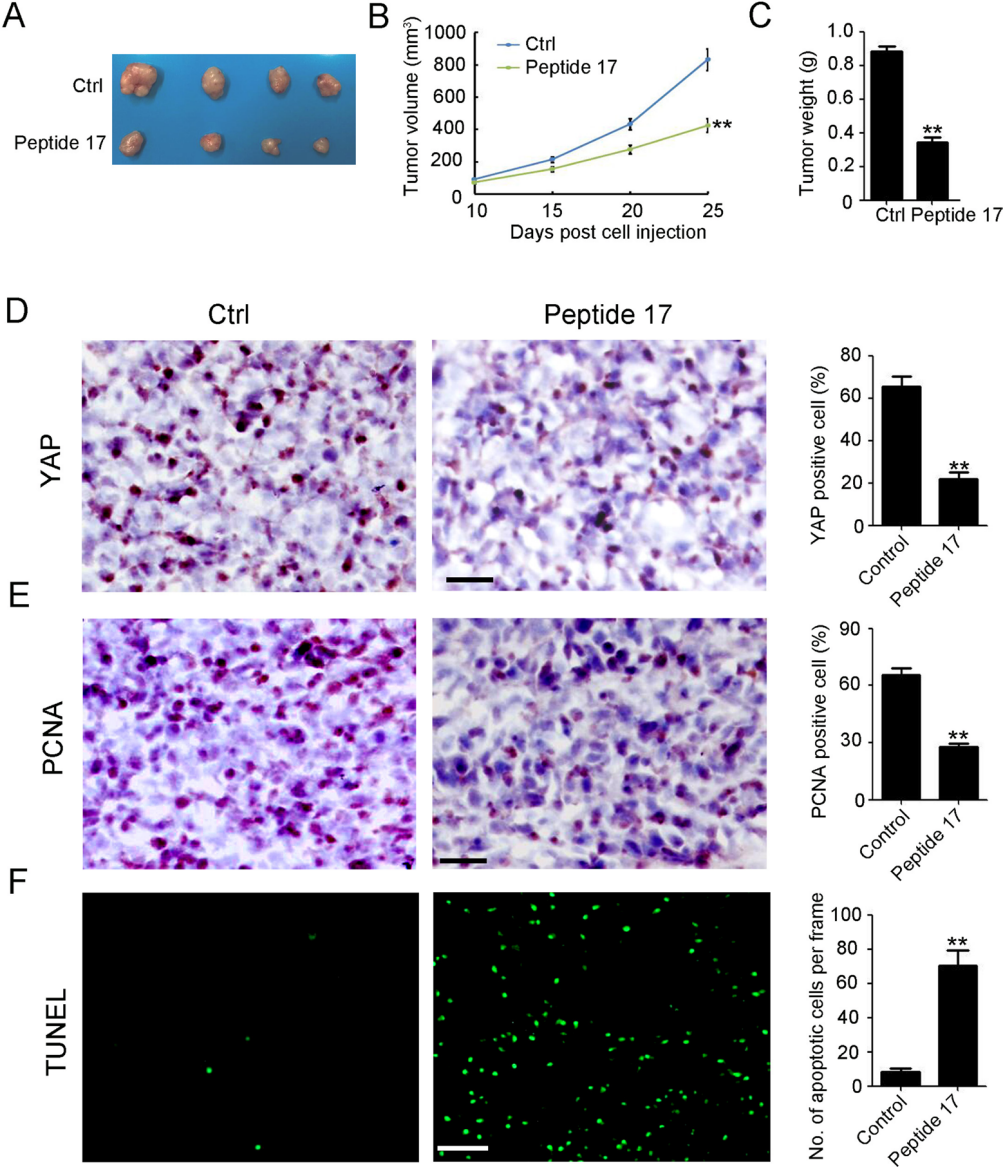
Knockdown of YAP inhibits neuroblastoma tumor growth *in vivo* (**A**) representative macroscopic findings of neuroblastoma tumors. (**B**, **C**) tumor volume (*n* = 4; **P < 0.01), and end-stage tumor weight (*n* = 4;**P < 0.01) after treatment of SH-SY5Y tumors with Peptide 17 or Control (Ctrl). (**D**, **E**) IHC staining of YAP and PCNA expression in SH-SY5Y tumors. The number of YAP and PCNA positive cells and total cells were counted in 5 random fields and analyzed (**P < 0.01). (**F**) TUNEL assay was performed to detect apoptotic cell in SH-SY5Y tumors. The number of apoptotic cells were counted in 5 random fields and analyzed (**P < 0.01).

To establish whether YAP was a potential tumor therapy target *in vivo*, SH-SY5Y cancer cells were injected into the flank of female wild-type (WT) BALB/c nude mice to establish a subcutaneous tumor model. Then, the specific inhibitor of YAP, Peptide 17, was utilized to treat the mice by intravenous injection [25]. As shown in Figure 3A, Peptide 17 significantly reduced tumor volume (Figure 3B) and weight (Figure 3C) by 49.2% and 61.1%, respectively. YAP staining confirmed that Peptide 17 efficiently inhibited YAP in SH-SY5Y tumors (Figure 3D). Peptide 17 also dramatically inhibited the proliferation of SH-SY5Y tumors as indicated by PCNA staining (Figure 3E). Additionally, more TUNEL-positive (apoptotic) cells were observed in the SH-SY5Y tumors after YAP inhibition (Figure 3F). Collectively, the *in vivo* data suggest that YAP is a potential therapy target for NB.

### Knockdown of YAP inhibits the proliferation and tumorigenesis of cisplatin-resistant NB

**Figure 4:**
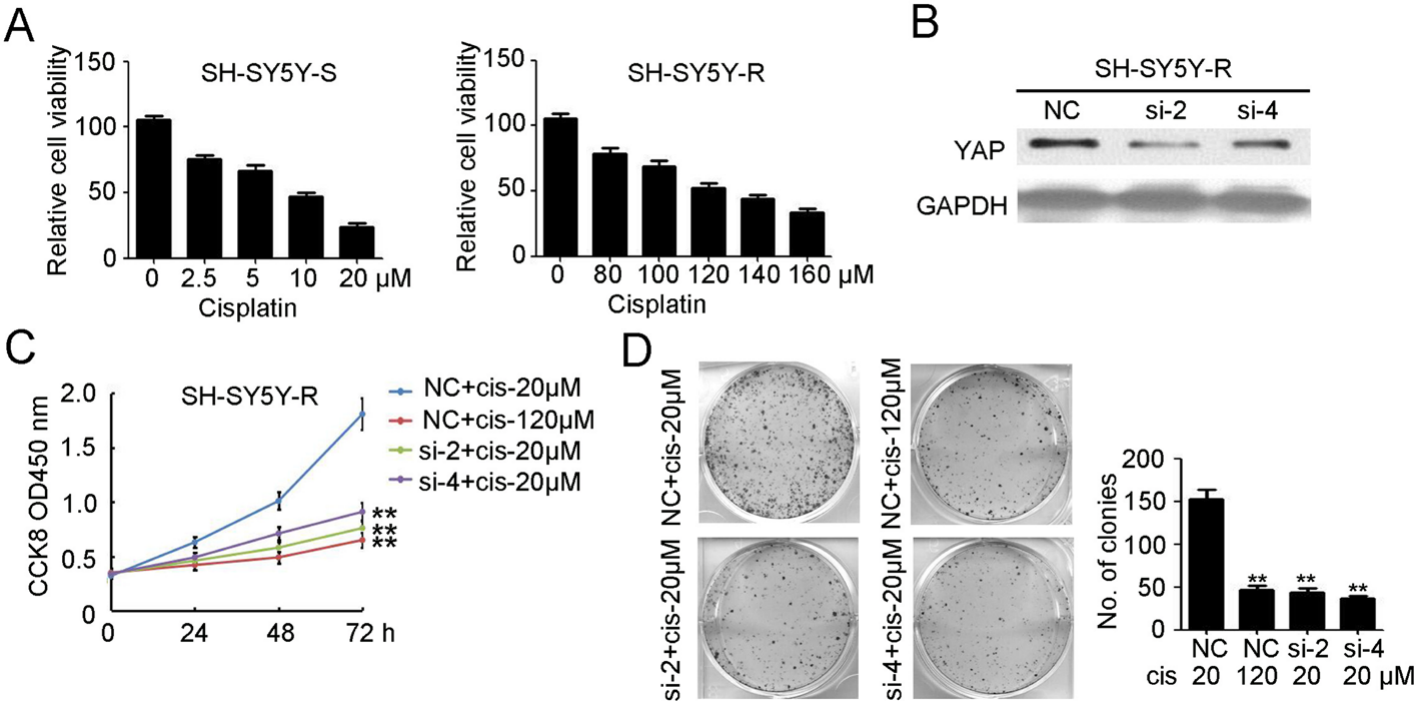
Knockdown of YAP inhibits the proliferation and tumorgenesis of cisplatin resistant neuroblastoma (**A**) cell counting kit-8 assay was performed to determine cell proliferation of selected cisplatin sensitive SH-SY5Y cells (SH-SY5Y-S) and cisplatin resistant SH-SY5Y cells (SH-SY5Y-R) under different concentration of cisplatin treatment. Data represent the means ± SD from three independent experiments (**P < 0.01). (**B**) siYAP-2 and siYAP-4 were used to transfected SH-SY5Y-R cells. Total cellular extracts were prepared and subjected to western blotting using antibody against YAP. GAPDH was used as a loading control. (**C**) cell counting kit-8 assay was performed to determine cell proliferation of SH-SY5Y-R cells with NC or siYAP-2/4 transfected, combined with 20 μM or 120 μM cisplatin treatment. Data represent the means ± SD from three independent experiments (**P < 0.01, compared with NC + cisplatin 20 μM group). (**D**) colon formation assay was performed to determine tumorgenesis of SH-SY5Y-R cells with NC or siYAP-2/4 transfected, combined with 20 μM or 120 μM cisplatin treatment. Colony number was counted and analyzed. Data represent the means ± SD from three independent experiments (**P < 0.01, compared with NC + cisplatin 20 μM group).

Next, cisplatin was used to treat SH-SY5Y cells and the cisplatin-resistant SH-SY5Y cells were selected and named SH-SY5Y-R. Viability assays indicated that the IC50 of cisplatin for SH-SY5Y-S (Figure 4A) and SH-SY5Y-R (Figure 4B) was approximately 10 μM and 120 μM, respectively. To explore whether YAP has an effect on cisplatin-resistant NB, we used siYAP-2 and siYAP-4 to transfect SH-SY5Y-R cells. Western blotting results indicated that siYAP-2 and siYAP-4 transfection efficiently inhibited the expression of YAP in SH-SY5Y-R cells (Figure 4C). Knockdown of YAP, combined with low-dose cisplatin treatment, significantly reduced the proliferation of SH-SY5Y-R cells (Figure 4D). Furthermore, fewer colonies were formed in the siYAP-2 and siYAP-4 transfected group, combined with low-dose cisplatin treatment (Figure 4E). Taken together, our results confirmed the involvement of YAP in maintaining cisplatin resistance in NB.

### Peptide 17 inhibits cisplatin resistant NB tumor growth *in vivo*

**Figure 5:**
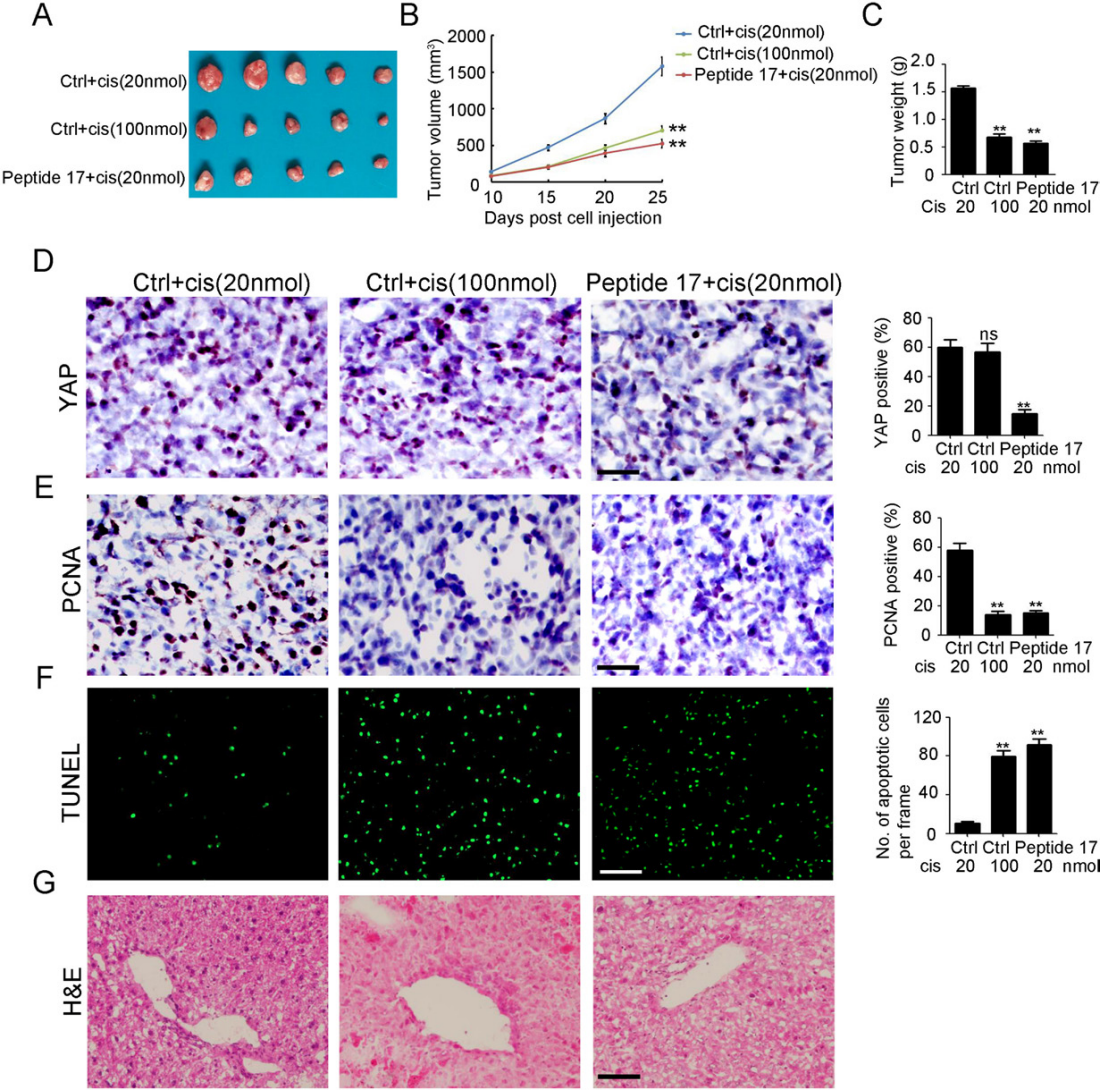
Knockdown of YAP inhibits cisplatin resistant neuroblastoma tumor growth *in vivo* (**A**) representative macroscopic findings of neuroblastoma tumors. (**B**, **C**) tumor volume (*n* = 4; **P < 0.01), and end-stage tumor weight (*n* = 4;**P < 0.01) after treatment of SH-SY5Y tumors with Peptide 17 or Control (Ctrl), combined with 20 nmol/day or 100 nmol/day cisplatin treatment. (**D**, **E**) IHC staining of YAP and PCNA expression in SH-SY5Y-R tumors. The number of YAP and PCNA positive cells and total cells were counted in 5 random fields and analyzed (**P < 0.01). (**F**) TUNEL assay was performed to detect apoptotic cell in SH-SY5Y-R tumors. The number of apoptotic cells were counted in 3 random fields and analyzed (**P < 0.01). (**G**) H&E staining of liver in nude mice after treatment.

To further investigate whether the inhibition of YAP enhances NB sensitivity to cisplatin *in vivo*, SH-SY5Y-R cells were injected into the flank of female wild-type (WT) BALB/c nude mice to establish a subcutaneous tumor model. As shown in Figure 5A, treatment of mice with high-dose cisplatin (100 nmol/day) considerably reduced tumor volume (Figure 5B) and weight (Figure 5C) by 55.8% and 56.5%, respectively. Meanwhile, injection of Peptide 17, combined with low-dose cisplatin (20 nmol/day) significantly inhibited tumor volume (Figure 5B) and weight (Figure 5C) by 61.6% and 63.7%, respectively. Injection of Peptide 17 significantly inhibited YAP expression in SH-SY5Y-R tumors whereas cisplatin had no observed effects on YAP expression (Figure 5D). Fewer proliferative cells were observed in both the high-dose cisplatin treatment group and Peptide 17 combined with low-dose cisplatin treatment group (Figure 5E). Additionally, more apoptotic cells were found in both of the above groups (Figure 5F). Notably, more severe liver injury was found in the high-dose cisplatin treatment group compared to that in the Peptide 17 combined with low-dose cisplatin treatment group (Figure 5G, 5H). Collectively, YAP is an efficient therapeutic target for cisplatin-resistant NB.

## DISCUSSION

Our study provides several significant lines of evidence to support that YAP modulates tumorigenesis and cisplatin resistance in NB. We found that YAP expression was upregulated in NB and correlated with poor tumor grading. In addition, knockdown of YAP by siRNA or Peptide 17 treatment significantly inhibited NB growth, tumorigenesis, and invasion both *in vitro* and *in vivo*. Furthermore, decreased YAP expression impaired the cisplatin resistance of NB and caused less liver toxicity to mice.

The Hippo pathway plays a key role in tissue homeostasis and organ size control by regulating tissue-specific stem cells [26]. Dysregulation of the Hippo pathway, especially molecules such as YAP and TAZ, which are the downstream transcriptional coactivators of the Hippo pathway, is associated with cancer development [27]. YAP is highly expressed in neural stem cells (NSCs), which are multipotent progenitors present in the nervous system [12]. In the developing neural tube of vertebrates, YAP is expressed by ventricular zone progenitor cells and co-localizes with Sox2, a neural progenitor marker [28, 29]. Overexpression of YAP in the neural tube leads to reduced neural differentiation and a marked increase in neural progenitor cell numbers due to accelerated cell cycle progression and recurring cell cycle exit [28, 29]. YAP has been demonstrated to be upregulated in glioma and increased the cell proliferation ability [30]. High expression of YAP correlated with poor prognosis of glioma patients [30]. Further functional study identified that inhibition of the YAP/TAZ-CTGF-Akt signaling axis by exogenous overexpression of Cbx7 induced cell death and inhibited cell proliferation, colony formation, and migration/invasion of the glioma cells [31]. In the present study, we first identified the high expression of YAP in NB. Consistently, YAP expression correlated with late staging of patients with NB. Knockdown of YAP significantly impaired NB growth, tumorigenesis, and invasion both *in vitro* and *in vivo*. Investigations into the underlying mechanism have found that the CTGF-Akt signaling axis and SOX-9 are impaired by YAP deletion in NB, which were the identified downstream targets of YAP [32, 33]. Previous study have demonstrated a significant reduction in tumor growth rate in a hepatocellular carcinoma xenograft model after introducing a YAP-like peptides, Peptide 17 [25]. These results lay a solid foundation for understanding the expression and function of YAP in NBs; however, further investigations are needed to determine the relationship between YAP and MYCN, the predictor for NB development [34–36].

Classical cytotoxic and genotoxic drugs, radio-therapeutic interventions, and newly designed anticancer agents targeting signaling and metabolic pathways are the most widely used therapeutic strategies directed against human solid tumors. The success of these strategies is based on their ability to disrupt cancer cell survival, invasive growth, tumor angiogenesis, and metastasis. Under treatment with these therapeutic strategies, multifactorial molecular and cellular mechanisms induced in both cancer cells and the stromal compartments of growing epithelial tumors may cause anticancer drug resistance [37]. Thus, anticancer drug resistance is the major obstruction for cancer therapy. YAP was found to be involved in modulating multidrug resistance in NSCLC [16], HCC [24], and colon cancer [23]. In the present study, we identified a novel function where YAP depletion significantly inhibited cisplatin-resistant NB growth and tumorigenesis *in vitro*. In the animal study, we also found that Peptide 17 dramatically prevented cisplatin-resistant NB growth, being as effective as high-dose cisplatin treatment. Notably, lesser liver injury was observed in the Peptide 17 treatment group compared to that in the high-dose cisplatin treatment group.

In conclusion, our study indicates that high expression of YAP correlates with advanced tumor staging and promotes tumorigenesis and invasion in NB. Inhibition of YAP impairs tumor growth and cisplatin resistance of NB. Our results identify YAP as a potential therapeutic target for cisplatin-resistant NB.

## MATERIALS AND METHODS

### Primary neuroblastoma tumors

In total, 38 pairs of diagnostic primary neuroblastoma tumor samples and adjacent tissues were obtained from the Department of Pediatric surgical oncology, Children's Hospital of Chongqing Medical University. Research was approved by the Research Ethics Committees of Chongqing Medical University. Written informed consent were signed by the parents or guardians of the pediatric patients. The tumor grade were identified according to clinical diagnosis.

### Cell culture and treatment

SK-N-SH and SH-SY5Y NB cell lines were purchased from American Type Culture Collection (ATCC, Manassas, VA, USA) and cultured in DMEM supplemented with 10% fetal bovine serum (FBS) and antibiotics at 37°C in a humid incubator with 5% CO_2_. The SH-SY5Y cells were maintained at the initial cisplatin concentration of 10 μM (IC50). The dose of cisplatin was titrated gradually to the final concentration of 80 μM after 6 weeks. The selected cisplatin resistant SH-SY5Y cells was named as SH-SY5Y-R cells, and the cisplatin sensitive SH-SY5Y cells was named SH-SY5Y-S cells. SH-SY5Y-R cells were established and then were maintained in DMEM medium with 10% FBS containing 80 μM cisplatin.

The siRNAs targeting YAP were purchased from Riobio (Guangzhou, Guangdong, China) and used to transfect SK-N-SH, SH-SY5Y and SH-SY5Y-R cells with riboFECTTM CP transfection reagent, following the instructions supplied by the manufacturer.

### Cell viability assay

Cells, after siRNAs transfection, were cultured in a 96-well plate. At 0, 24, 48 and 72 hours post transfection, CCK8 was added into the plate well and incubated at 37°C for 4 hour. The OD450 nm was measured by microplate reader. SH-SY5Y-S and SH-SY5Y-R cells were cultured in a 96-well plate and treated with different doses of cisplatin. The cell viability were also determined by CCK8 assay.

### Western blotting

For protein extraction, the cell lines were lysed by using RIPA lysis buffer (Beyotime, Beijing, China) contained 1% protease inhibitor cocktails (Roche, Lewes, UK). The concertation was determined by BCA assay (Beyotime, Beijing, China). The total amount of protein for each sample was 20 μg, and the samples were run on 10–12% gradient SDS–polyacrylamide gels and then were transferred to polyvinylidene fluoride membranes (Millipore, MA, USA). After blocking with 5% non-fat milk in TBS/T, the membranes were probed with primary antibodies YAP, CTGF, SOX-9, PTEN, p-Akt and Akt (Cell Signaling Technology, Inc) and GAPDH (Santa Curz Inc) in 4 °C overnight. The membranes were then incubated with appropriate second antibodies at room temperature for 1 hour, and finally were detected by using an ECL blotting analysis system (Millipore, Bellerica, MA). The relative expression was determined by Image J software.

### Colony formation assay

1000 SK-N-SH and SH-SY5Y cells after transfection were added into the 6 well plate, was fixed with DMEM medium containing 10% FBS. 7–10 days later, the plate was fixed with 4% paraformaldehyde and stained with crystal violet (Beyotime, Beijing, China). The number of colony formation were counted and analyzed.

### Invasion assay

After dilution with DMEM medium (1:5), Matrigel was added into a 8.0 μm transwell (BD). 30 mins later, 2 × 10^4^ SK-N-SH and SH-SY5Y cells after transfection were added into the upper well containing serum-free medium. The below well was fixed with DMEM medium containing 10% FBS. 24 hours later, the transwell was fixed with 4% paraformaldehyde and stained with crystal violet (Beyotime, Beijing, China). The invaded cells were counted and analyzed.

### Animal study

Female BALB/c nude mice were purchased from the Model Animal Research Center of Nanjing University (Nanjing, China) and allowed to acclimate for 1 week before use. Studies were performed in accordance with institutional guidelines concerning animal use and care. All mouse care and experiments were carried out in accordance with institutional guidelines concerning animal use and care of Chongqing Medical University. Human SH-SY5Y and SH-SY5Y-R xenografts were established by subcutaneously inoculating 5 × 10^6^ cells into nude mice. When the tumors reached a mean group size of 100 mm^3^, the mice were randomized into control and treatment groups to receive the following daily treatments for 14 days: Peptide 17 (0.2 mg/kg), low dose cisplatin (20 nmol/mice) and high dose cisplatin (100 nmol/mice). Tumor volume (V) was calculated as V = (length × width^2^ × 0.52). At the 25 days after tumor cell injection, the mice were sacrificed. The tumor were peeled off and weighted.

### Immunostaining and H&E staining

Immunostaining was performed as previous study indicated [38]. Briefly speaking, the tumor tissues were routinely fixed in 10% paraformaldehyde and embedded in paraffin. The 4 μm sections were dewaxed using xylene and rehydrated in graded alcohols. After antigen retrieval and blocking with goat serum, the sections were incubated overnight with the primary antibodies (YAP and PCNA, which is the marker of cell proliferation) at 4°C. The sections were incubated for 60 min at room temperature with labeled-dextran polymer (Zsbio, Beijing, China). The sections were developed with activated 3′-diaminobenzidinetetrahydrochloride (DAB) (maixin bio, Fuzhou, China) for 1–3 mins. The positive cells exhibited the deposition of brown DAB precipitate. The cell nuclei were stained with hematoxylin (Beyotime, Beijing, China). The positive cells were counted and analyzed.

The apoptotic cell were detected by the TUNEL assay (Millipore, MA, USA). All of the operations were followed the instructions supplied by the manufacturer. The localized green fluorescence of apoptotic cells from the fuorescein-12-dUTP was detected by fluorescence microscopy. The cell nuclei was stained by DAPI (Beyotime, Beijing, China). The apoptotic cells in 4–6 random fields were counted and analyzed.

The liver tissues were routinely fixed in 10% paraformaldehyde and embedded in paraffin. The 4 μm liver sections were dewaxed using xylene and rehydrated in graded alcohols. Then, Hematoxylin and Eosin were purchased from Beyotime (Beijing, China) and performed to stain cell nucleus and cytoplasm in turn followed the instructions supplied by the manufacturer.

### Statistical analysis

All experiments were repeated three to five times, and the data were expressed as the mean ± s.d. Statistical analysis was performed by the Student's *t*-tests for comparing two groups and by analysis of variance for multiple group comparisons; *P* < 0.05 was considered statistically significant.
