# Correction: YAP promotes tumorigenesis and cisplatin resistance in neuroblastoma

**DOI:** 10.18632/oncotarget.28138

**Published:** 2022-06-01

**Authors:** Chao Yang, Juan Tan, Jun Zhu, Shan Wang, Guanghui Wei

**Affiliations:** ^1^Department of Pediatric Surgical Oncology, Children’s Hospital of Chongqing Medical University, Ministry of Education Key Laboratory of Child Development and Disorders, Chongqing, China; ^2^China International Science and Technology Cooperation Base of Child Development and Critical Disorders, Chongqing, China; ^3^Chongqing Key Laboratory of Pediatrics, Chongqing, China; ^4^Clinical Department of Children’s Hospital of Chongqing Medical University, Lijia Campus, Chongqing, China; ^5^Department of Pathology, Children’s Hospital of Chongqing Medical University, Ministry of Education Key Laboratory of Child Development and Disorders, Chongqing, China; ^6^Department of Urology, Children’s Hospital of Chongqing Medical University, Ministry of Education Key Laboratory of Child Development and Disorders, Chongqing, China

**This article has been corrected:** In Figure 3D, the wrong picture was mistakenly selected for the IHC staining image of YAP in the Ctrl group (1st panel). The corrected Figure 3D, produced using the original data, is shown below. The authors declare that these corrections do not change the results or conclusions of this paper.

Original article: Oncotarget. 2017; 8:37154–37163. 37154-37163. https://doi.org/10.18632/oncotarget.16209

**Figure 3:**
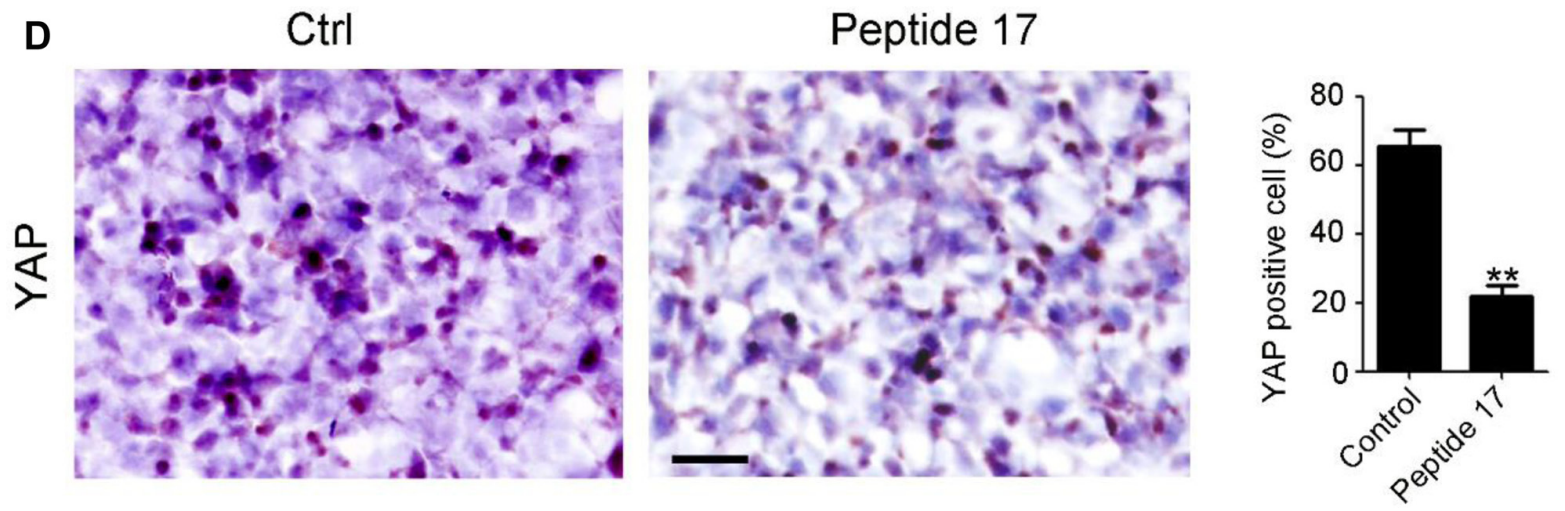
(**D**) IHC staining of YAP and PCNA expression in SH-SY5Y tumors. The number of YAP and PCNA positive cells and total cells were counted in 5 random fields and analyzed (^**^ *P* < 0.01).

